# How Single-Cell Transcriptomics of *Hydractinia* Is Informing the Evolution of Cnidarian Sensory Systems

**DOI:** 10.1093/icb/icaf090

**Published:** 2025-06-12

**Authors:** Christine E Schnitzler, Jingwei Song, Danielle de Jong

**Affiliations:** Whitney Laboratory for Marine Bioscience, University of Florida, St. Augustine, Florida, 32080, USA; Department of Biology, University of Florida, Gainesville, Florida, 32611, USA; Whitney Laboratory for Marine Bioscience, University of Florida, St. Augustine, Florida, 32080, USA; Department of Biology, University of Florida, Gainesville, Florida, 32611, USA; Coastal Oregon Marine Experiment Station (COMES), Oregon State University/USDA-ARS Pacific Shellfish Research Unit, Newport, OR 97365, USA; Whitney Laboratory for Marine Bioscience, University of Florida, St. Augustine, Florida, 32080, USA; Department of Biology, University of Florida, Gainesville, Florida, 32611, USA

## Abstract

For over a century, the colonial cnidarian *Hydractinia* has been employed as a research organism to study stem cells, germ cells, regeneration, and coloniality. For the last 70 years, it has also been used in allorecognition research within the field of comparative immunology. More recently, other aspects of *Hydractinia* biology, including sensory biology, have been explored. *Hydractinia* colonies are composed of a limited number of repeating structural units: polyps and the stolon tissue that connects polyps. Polyps are divided into three major types used for feeding, reproduction, or defense. Clonal lines grown in the lab provide unlimited material from a single genetic unit. Colonies have separate sexes and spawn regularly with exposure to light. Recently, genomic and transcriptomic resources have been released for two species of *Hydractinia: H. symbiolongicarpus* and *H. echinata*. Tools for gene expression manipulation have been developed for this organism, including CRISPR/Cas9 knockout, shRNA knockdown, and overexpression via synthetic RNA. Fluorescent transgenic reporter lines have been created via random integration of circular DNA plasmids and CRISPR/Cas9-mediated gene knockin. We recently constructed an updated single-cell transcriptomic atlas of adult *Hydractinia* colonies to explore the cellular biology and cell-type expression profiles of the animal. We are investigating known and novel cell types and validating spatial expression patterns of cell-type specific markers to enable further understanding of the animal’s cellular biology. This includes gaining a deeper understanding of the genetic control of cell differentiation of specific cell types from progenitor populations and uncovering the diversity of transcriptional subtypes that may be relevant to specific functions. Since *Hydractinia* is a model for whole-body regeneration, the identification and validation of new cell type and cell state markers will now allow for the elucidation of potential pathways involved in regenerating specific cell types, including testing alternative pathways for regeneration that include dedifferentiation and transdifferentiation. *Hydractinia* is poised to become a model for sensory biology research, as we can now fully explore their sensory cell types, including cnidocytes and neurons, and the expression and evolution of their gene complement with modern approaches and tools.

## Introduction

**Fig. 1:**
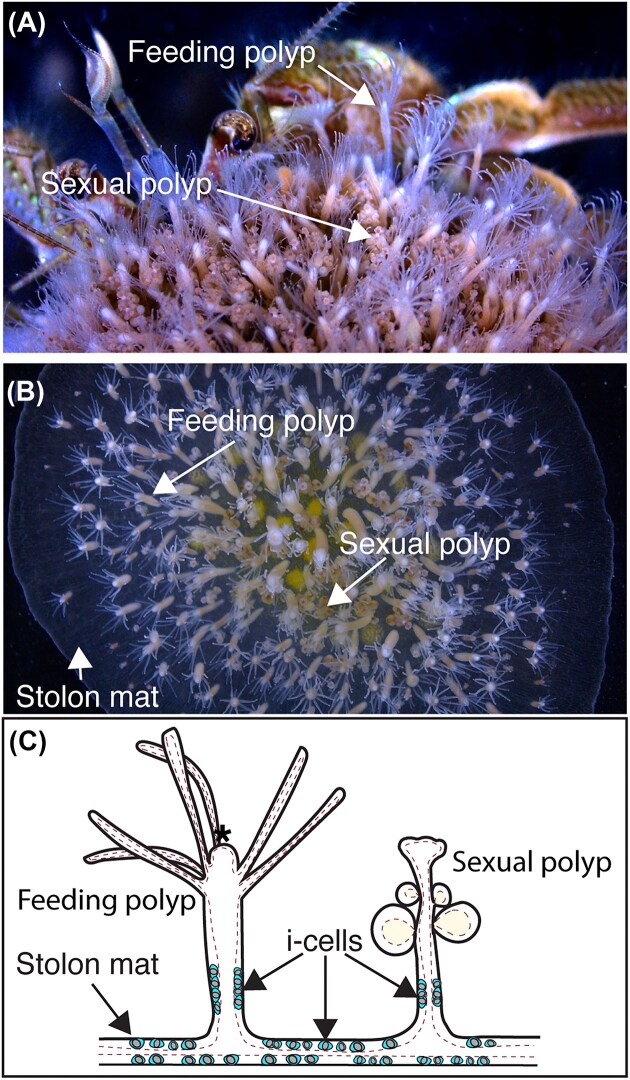
Overview of the *Hydractinia* colony, highlighting the two most common polyp types, feeding and sexual polyps. (A) *Hydractinia* colony on a shell inhabited by a hermit crab. (B) *Hydractinia symbiolongicarpus* colony growing on a glass microscope slide in the laboratory. This top-down view shows the distribution of feeding and sexual polyps growing from the stolon mat. (C) A schematic showing the basic morphology of feeding and sexual polyps growing from the stolon mat, highlighting the location of the i-cells. Asterisk indicates the location of the mouth of the feeding polyp.

*Hydractinia* is a colonial cnidarian in the class Hydrozoa. In nature, many *Hydractinia* species are found growing on the surface of shells inhabited by hermit crabs and are facultative symbionts, meaning they can live with or without the crab, although the relationship has been shown to benefit both (Fig. 1A) (Williams and McDermott 2004). Some *Hydractinia* species colonize other substrates like snails, crab carapaces, or rocks. In the late 1800s and early 1900s, studies emerged on colonial hydrozoans, including *Hydractinia*. August Weismann coined the terms “stem cells,” “germ cells,” and “germline” in an 1883 study of 37 colonial hydroids, essentially making them the original stem cell study animals (Weismann 1883). *Hydractinia* possess migratory adult pluripotent stem cells known as interstitial stem cells or “i-cells.” I-cells are found in specific locations of the colony, including in a “band-like” region in the ectoderm in the mid-gastric region of feeding polyps and sexual polyps, and throughout the ectoderm of the basal stolon mat and stolons that connect the polyps, but not at the distal tips of stolons (Müller 1964; Plickert et al. 2012; Bradshaw et al. 2015; DuBuc et al. 2020).

I-cells constantly self-renew, as well as generate all somatic and germ cell lineages of the animal as necessary, and contribute to their remarkable abilities to regenerate (Müller 1964; Müller et al. 2004; Künzel et al. 2010; Bradshaw et al. 2015; DuBuc et al. 2020; Varley et al. 2023). Early studies of regeneration were mostly observational and descriptive but document the impressive regenerative abilities and unique features of *Hydractinia* colonies (Peebles 1900; Hazen 1902). *Hydractinia* can regenerate most tissues and structures at different life stages, including in larvae (Schwoerer-Böhning et al. 1990) and in different polyp types of colonies (Peebles 1900). Within 24 h following feeding polyp head decapitation, a blastema—a region of highly proliferative i-cells that expresses conserved stem cell markers such as *Piwi1, Vasa*, and *Pl10*—is formed, and the head fully regenerates within 48–72 h (Bradshaw et al. 2015). EdU pulse-chase experiments showed that the i-cells that form the blastema migrate from the lower polyp body column (Bradshaw et al. 2015). When *Piwi1, Vasa*, or *Pl10* were knocked down following decapitation, regeneration was severely inhibited, but no effect on cell proliferation or blastema formation was observed. This suggests that this particular set of i-cell genes is not involved in controlling initial proliferation stages but rather plays a role in proceeding to differentiation and ultimately, tissue regeneration (Bradshaw et al. 2015). In addition, in certain tissues that appear to lack *Piwi1*^+^ i-cells, such as isolated oral tips of heads (hypostomes), differentiated somatic cells can undergo reprogramming to become i-cells before regenerating a new polyp (Salinas-Saavedra et al. 2023). This cellular plasticity and flexibility of cellular identity under different conditions is a feature that has only begun to be fully explored in *Hydractinia*. Recent studies of i-cells and their contributions to homeostasis and regeneration are building on these foundations to further establish *Hydractinia* as a model organism for stem cell biology and regeneration research (Quiroga-Artigas et al. 2022; Varley et al. 2023; Waletich et al. 2024; Curantz et al. 2025).

*Hydractinia* colonies are gonochoric and exhibit a division of labor where different adult polyp types have various functions—feeding, reproduction, and defense. There are specific names for these polyp types—gastrozooids for feeding, gonozooids for reproduction, and both dactylozooids, and tentaculozooids for defense (Cartwright 2003). All polyps are connected to one another through a basal mat comprised of endodermal stolons that form a shared gastrovascular canal with the polyps, sandwiched between upper and lower epithelial layers (Cartwright 2003). Chitinous defensive structures called spines protrude upwards from the basal mat of mature colonies. In the lab, colonies can grow on a variety of surfaces, such as glass microscope slides, although defensive polyps are rare or absent in animals cultured in the lab, perhaps due to the lack of predators or disturbance from other organisms (Collcutt 1897; Fig. 1B). Colonies release gametes during light-induced broadcast spawning events. Zygotes develop into planula larvae that typically attach to hermit crab-occupied shells based on the presence of a bacterial biofilm (Yund et al. 1987; Rischer et al. 2022). Settled larvae undergo metamorphosis to a primary polyp in response to specific cues from the bacterial biofilm (Rischer et al. 2022). It has been shown that two structurally different, bacterium-derived metabolites—specific lysophospholipids and exopolysaccharides—converge to induce metamorphosis of *Hydractinia* larvae into a primary polyp (Guo et al. 2021). This primary polyp will extend stolons from its aboral end, which will in turn bud new polyps, eventually forming a colony consisting of different polyp types (Müller and Buchal 1973). A colony will completely cover its shell or other substrate if conditions permit.

When two or more colonies are growing on the same shell, if they are unrelated conspecifics, eventual contact between them will elicit a well-characterized agonistic response, known as allorecognition, where competition leads to different outcomes (Buss et al. 1984). This ability to discriminate self from unrelated non-self has made *Hydractinia* a study system for the evolution of allorecognition (Lange et al. 1989; Mueller and Rinkevich 2020). Histocompatibility in *Hydractinia* has a genetic basis that has been mapped to one large region of the genome called the allorecognition complex (ARC) containing polymorphic allodeterminants *Allorecognition 1* (*Alr1*) and *Allorecognition 2* (*Alr2*), as well as at least 41 additional *Alr*-like genes (Mokady and Buss 1996; Nicotra et al. 2009; Huene et al. 2022). As the complexity of the ARC region and the diversity of genes involved are now starting to be recognized, further studies will help uncover whether the *Hydractinia* allorecognition system shares an evolutionary origin with similar systems in other marine invertebrates (Nicotra 2022).

Relatively few studies have been carried out on the topic of sensory biology in *Hydractinia*, with many such studies focused on the larval life stage (Weis et al. 1985; Katsukura et al. 2003; Plickert and Schneider 2004; Seipp et al. 2010; Birch et al. 2024), and a smaller number focused on the adult phase (Stokes 1974; Schmich et al. 1998; Chrysostomou et al. 2022). In this paper, we review what is known about the major cell types in *Hydractinia*, focusing on sensory cell types like cnidocytes and neurons. We then present an updated view of these cell types in adult polyps by drawing from our single-cell transcriptomic analyses, from which we derived cell-type specific marker genes to visualize these cells *in situ*. We focus our analyses first on the two major cnidocyte types in *Hydractinia* polyps (desmonemes and euryteles) and second on two transcriptionally distinct neuron types with non-overlapping patterns in polyps that likely are not exclusive groupings of either ganglion or sensory neurons. These efforts to characterize the different cnidocyte and neuron types in a model hydroid will benefit the study of cnidogenesis, neurogenesis, and the regeneration and replacement of these fundamental cell types, allowing for a more complete understanding of this remarkable regenerator and how it compares to other animals with similar abilities.

### Cnidarian sensory systems

Cnidarian sensory systems include sensory cell types and structures that range from fully functional eyes and rhopalia in jellyfish, to defensive “battery cell” complexes in *Hydra* tentacles, to sensory neurons and cnidocytes found in nearly all cnidarian species (Hobmayer et al. 1990; Jacobs et al. 2007; Katsuki and Greenspan 2013; Picciani et al. 2018). The evolution of these cells and structures and their relationship to bilaterian sense organs and structures has been explored, and hypotheses about the relationship of these unique features to those in the common ancestor of cnidarian and bilaterians have been previously generated (Jacobs et al. 2007; Picciani et al. 2018). Here, we focus on *Hydractinia symbiolongicarpus*, a species possessing the typical set of sensory cells but a seeming lack of organized sensory structures. We are utilizing an updated *Hydractinia* adult single-cell atlas (Song et al. 2025) to identify and explore cell clusters, to identify genes that are uniquely expressed in these clusters, and to perform spatial expression studies of cell-type markers to better understand the organization and function of these cells in the adult animal. This set of information will help build a foundation that will ultimately contribute to a better understanding of the evolution of cnidarian sensory cells and systems by allowing for comparisons with other well-studied cnidarians. Understanding the diversity of cell types and cell-type transcriptional profiles in a variety of animals will help with identifying both conserved evolutionary processes and drivers of cell-type diversification in different lineages and the building blocks for sensory structures. It will be informative to see what conserved gene sets are found across a wide array of animal sensory systems and what leads to more complex sensory structures such as rhopalia—complex sensory organs found in jellyfish that contain light-sensitive cells, which are sometimes specialized into eyes, as well as neurons and statocysts. In *Hydractinia* specifically, research into regenerative mechanisms will be aided by providing a starting point for deeper understanding of how sensory cells are replaced following injury, as we now have markers for specific cell types that can be used to track their development and replacement.

### *Hydractinia* cell types

All cnidarians have two cell layers: the outer ectoderm (epidermis) and the inner endoderm (gastrodermis) separated by a gelatinous extracellular matrix known as the mesoglea. In addition, there are several cell types described in *Hydractinia* beyond i-cells; these include epithelial cells (both ectodermal and endodermal), epitheliomuscular cells, zymogen and mucous gland cells, nematocytes (stinging cells), as well as ganglion and sensory neurons (Campbell 1967; Stokes 1974; Weis and Buss 1987). Some cell types, such as the atrichous isorhiza cnidocytes and special neurosensory cells, are specific to the larval stage of *Hydractinia* (Weis et al. 1985). Recent studies in the hydrozoans *Hydra, Clytia*, and *Hydractinia* have begun to identify cell-type marker genes for many of these described cell types, as well as identifying cell differentiation states and various subtypes via single-cell RNA sequencing (Siebert et al. 2019; Chari et al. 2021; Cazet et al. 2023; Schnitzler et al. 2024). Trajectories of cellular differentiation for certain cell types have been recovered in some hydrozoan cell atlases, such as the one for *Hydra vulgaris*, where adult animals are constantly turning over their cells (Campbell 1967; Campbell and David 1974; Holstein et al. 1991; Siebert et al. 2019; Primack et al. 2023). This occurs to some extent in *Hydractinia*, where cnidocytes and neurons are frequently made under homeostatic conditions; however, epithelial cells and gland cells may turn over less frequently, a phenomenon that was reflected in the first single-cell atlas of the adult animal (Schnitzler et al. 2024). Single-cell atlases are also enabling detailed studies of cell types and cell states by combining the identification of specific marker genes from these atlases with modern tools that can be used to determine where these genes are expressed *in situ*. For example, multi-color HCR-FISH is now allowing visualization of several cell-type markers simultaneously to examine the spatial location of multiple cell types (Choi et al. 2018). Fluorescent transgenic cell-type specific reporter lines generated for *Hydractinia* have also enabled *in vivo* imaging of cellular processes and cell migration, helping to determine the differentiation potential and contribution of stem cells to tissues in different contexts, including polyp head regeneration, germ cell development, and the potency of a single transplanted i-cell (Künzel et al. 2010; Bradshaw et al. 2015; Dubuc et al. 2020; Varley et al. 2023). As modern tools for cellular biology continue to develop and evolve, further insights will be discovered regarding cnidarian cell-type evolution and function.

### Cnidocytes

**Fig. 2:**
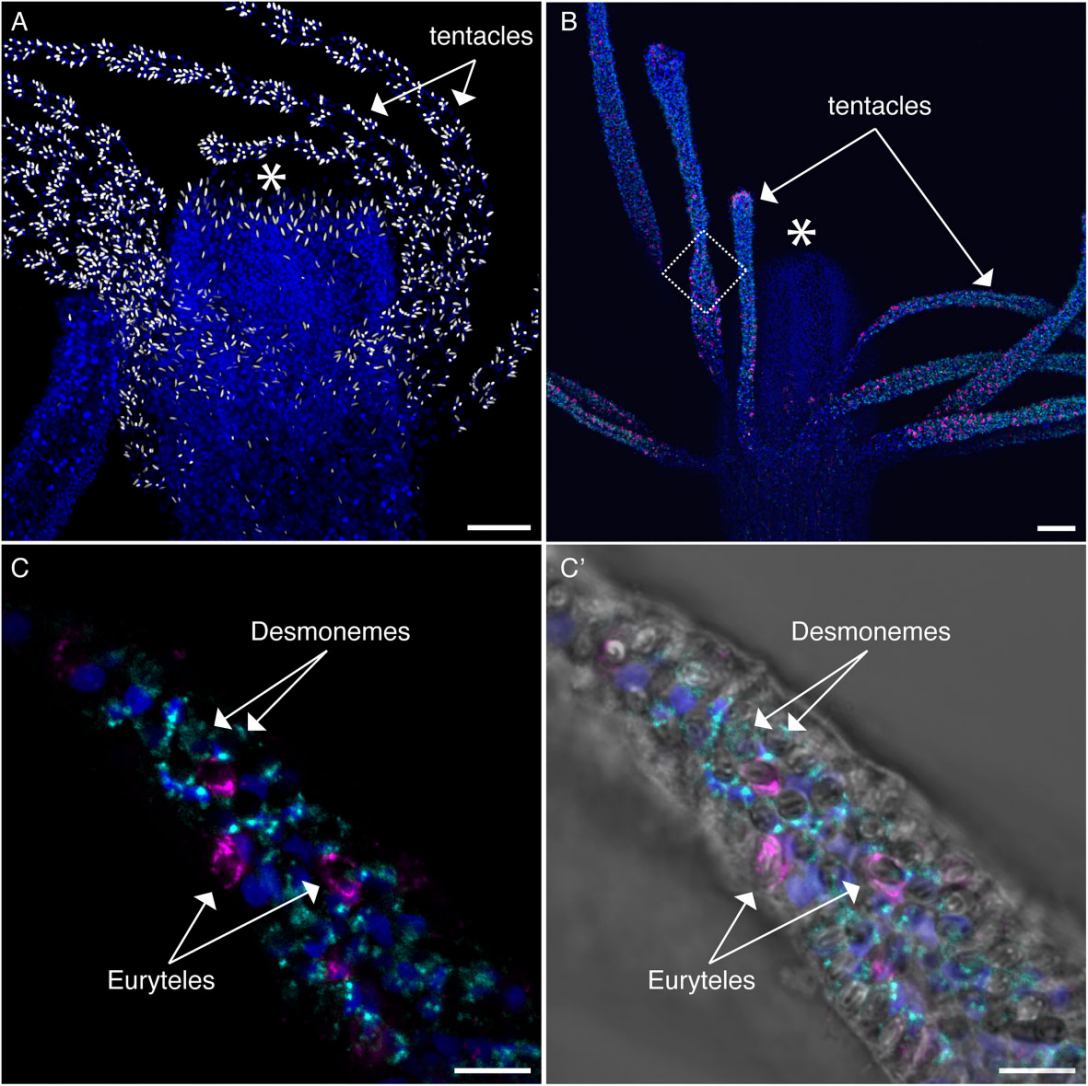
Cnidocytes in the *Hydractinia* feeding polyp. (A) High-concentration DAPI staining shows the distribution of cnidocyst-capsule containing cnidocytes in the oral half of a feeding polyp, where mature cnidocytes (in white) are densely packed in the tentacles, and less dense in the hypostome and body column. (B) Double HCR-FISH of two mature cnidocyte markers (HyS0002.245 in cyan, HyS0027.82 in magenta) shows spatial location in the oral half of a feeding polyp. (C) Zoom-in of a single tentacle showing location of putative desmonemes and euryteles. (C’) Zoom-in showing the two mature cnidocyte markers together with DIC to highlight cnidocyte capsules. Nuclei stained with Hoechst are shown in dark blue. Scale bars are 50 µm in A and B and 10 µm in C and C’. Asterisk in A and B indicates the mouth.

Cnidocytes, cnidarian-specific stinging cells, are a specialized cell type (Tardent 1995). They are mechanosensory cells classified as a modified neural cell type based on many lines of evidence, including neurophysiological properties, ultrastructural features, and expression of neural genes in cnidoblasts (Thurm et al. 2004; Galliot et al. 2009; Rentzsch et al. 2017). Cnidocytes are made up of an external sensory cilium called a cnidocil, and an internal cnidocyst organelle—a pressurized capsule that houses a coiled, barbed, harpoon-ended, thread-like tubule structure—that is extruded when discharged upon stimulation of the sensory cilium (Özbek et al. 2009). Once discharged, the tubule can ensnare, inject venom into, or adhere to its target, allowing for both prey capture and defense from predators. Cnidocytes include many specific subtypes, largely defined morphologically by their tubule structure (Fautin 2009). High-concentration DAPI staining of cnidocyst capsules (Szczepanek et al. 2002) provides a visualization of the spatial distribution of these cells throughout *Hydractinia* colonies and highlights the fact that the tentacles and hypostome of *Hydractinia* adult and juvenile polyps are completely packed with poly-gamma-glutamate^+^ cnidocytes (Fig. 2A). Capsule-staining cnidocytes are also present in the feeding polyp body column (albeit with lower density), in other polyp types, and throughout stolon tissue (not shown).

There are two major cnidocyte types in adult *Hydractinia* colonies—desmonemes and euryteles. These cell types are distinguished by size differences (desmonemes are smaller, euryteles larger), but also by different cnidocyst capsule morphologies (Mills 1976; Lange et al. 1989; Schuchert 2014). A previous study found that within feeding polyp tentacles, desmonemes were the predominant type comprising about 75% of the cnidocytes, whereas small euryteles only comprised 25% of the cnidocytes (Klompen et al. 2022). The area around the hypostome of these polyps exclusively contained euryteles (Klompen et al. 2022). Desmonemes primarily entangle prey by wrapping their thread around the target and do not contain or inject any toxins, while euryteles often pierce prey and are thought to contain toxins (Klompen et al. 2022). Feeding behavior in *Hydractinia* supports the need for these two cell types in that feeding polyps first ensnare prey in their tentacles presumably with desmonemes, then often paralyze them likely with toxins from euryteles, followed by bringing the prey to their mouth, where additional euryteles are likely discharged and toxins are injected to further paralyze or kill the prey item just prior to ingestion (Klompen et al. 2022). Our updated adult single-cell transcriptomic atlas (see the “Materials and methods” section) shows a trajectory of cnidogenesis starting from the single population of i-cells that diverge into two separate endpoints (Song et al. 2025). To examine if the endpoints represent the two fully differentiated major cnidocyte types, we selected marker genes associated with each (HyS0002.245 and HyS0027.82) and determined their spatial location in feeding polyps. HyS0002.245 is annotated as “uncharacterized,” and HyS0027.82 has multiple thrombospondin-1 (TSP_1) domain repeats. HyS0027.82 also has BLAST hits to “SCO-spondin-like” and “coadhesin” genes in other animals, including corals, where coadhesin may function in cell adhesion within the coral skeleton (Drake et al. 2020). We found that these two marker genes were expressed in non-overlapping populations in the tentacles of *Hydractinia* feeding polyps (Fig. 2C and C’). Close examination of the cnidocyst structures of these cells under differential interference contrast (DIC) illumination, which makes transparent structures visible that are difficult to see using brightfield illumination, indicated that they appear to be aligned with desmonemes (HyS0002.245) and euryteles (HyS0027.82) (Östman 2000; Klompen et al. 2022). Although we did not quantify cell numbers, we found a similar general pattern of desmonemes being predominant in tentacles consistent with previous results (Klompen et al. 2022), with some variation (Fig. 2). It is likely that the two endpoint marker genes only highlight a subset of differentiated cnidocyte types, as certain tissue regions that clearly have cnidocytes, such as in the hypostome (Fig. 2A), where small euryteles are known to be present, are not marked by either gene. It may be that these hypostome euryteles contain a distinct transcriptional profile or that the single gene marker we chose for euryteles (HyS0027.82) is not expressed in this subset of euryteles. The single-cell atlas provides a promising resource for future identification of additional cnidocyte subtypes.

### Neurons

In cnidarians, beyond cnidocytes, which are considered a modified neural cell type, the concept of neurons relates to two related groups of cells—ganglion cells and sensory (or sensory-motor) cells, both of which have neurites and establish synapses with other cells (Rentzsch et al. 2017). Ganglion cells have cell bodies found in a “deep, basi-epithelial position,” are often equated to interneurons, can help coordinate information along the body, and can make synapses with muscle cells or cnidocytes to guide muscle contraction or cnidocyte discharge among other functions (Rentzsch et al. 2017). Sensory cells are described as having elongated cell bodies and always bear an apical cilium that is said to emerge from the body of the animal. This cilium functions to detect and convert environmental stimuli into electrical signals, which are then transmitted to other cells (Rentzsch et al. 2017). Similar to cnidocytes, there are multiple subtypes for each group of neurons defined by their function (mechanosensory or chemosensory), by morphology (pattern and number of neurite projections), by molecular features (neurotransmitter or other gene expression) or a combination of these traits (Rentzsch et al. 2017). In the recent era of transgenic reporter lines and single-cell RNAseq datasets, our understanding of these neural subtypes is being further developed (Rentzsch et al. 2019; Siebert et al. 2019; Primack et al. 2023).

Ganglion and sensory neurons were first identified and defined in *Hydractinia echinata* with light and electron microscopy (Stokes 1974). This study described ganglion neurons as typically having microtubules and electron dense vesicles, being mostly bipolar, with cell bodies measuring from 6 to 9 µm in length and up to 3.5 µm in width, with longer neurite projections extending from the cell body. Stokes found ganglion neurons distributed throughout feeding polyps, with a higher density in the body column and tentacles, a lower density in the hypostome, and many present throughout the upper ectoderm of the stolon but not at stolon tips. Stokes described sensory neurons as bearing a single ciliary process at the apical end of the cell and microtubules with varying sizes based on location. These sensory cells were 7–10 µm in length and 4–6 µm wide in tentacles and hypostome and 8–12 µm in length and 2.5–4 µm wide at the base of feeding polyps and in the basal mat tissue. Sensory cells were said to be common in the hypostome of feeding polyps and located along the entire length of tentacles but particularly concentrated at tentacle tips. Sensory cells were also found to be present in the body column oriented perpendicular to the longitudinal axis (as they were in the hypostome and tentacles), but they were not as well developed here, and their apical cilium did not penetrate the body wall. Finally, sensory cells were quite rare in the basal mat. This study noted that there is only an ectodermal nerve net in *Hydractinia*, in contrast with *Hydra*, which bears both an endodermal and an ectodermal nerve net. A decade later, an ultrastructure study on metamorphosing polyps described both “nerve cells” and “sensory cells” in the ectoderm of larvae but only described generic “nerve cells” in young primary polyps (Weis and Buss 1987) rather than labeling them as either ganglionic or sensory.

A neuropeptide antibody study of *H. echinata* showed *RFamide* and *GLWamide* positive neurons in different subsets of ectodermal neurons in young feeding polyps with *RFamide*^+^ neurons highly concentrated in the hypostome tissue and *GLWamide*^+^ neurons concentrated at the base of the tentacles (Schmich et al. 1998). In older polyps, a dense longitudinal arrangement of *GLWamide*^+^ fibers was shown throughout the polyp body column (Schmich et al. 1998). An updated study using *H. symbiolongicarpus* (Chrysostomou et al. 2022) provided immunofluorescence images of those same two neuropeptides and included a representative schematic of this staining (see Fig. 1—Fig. supplement 4 from that study) showing the location of three putatively different neuron types in *Hydractinia* feeding and sexual polyps: *GLWamide*^+^ ganglionic neurons, *RFamide*^+^ ganglionic neurons, and *RFamide*^+^ sensory neurons. In this schematic, *GLWamide*^+^ ganglionic neurons were shown mainly at the base of the tentacles, extending through the feeding polyp body column; *RFamide*^+^ ganglionic neurons were depicted as the only neuron type in the tentacles of feeding polyps, and *RFamide*^+^ ganglionic and sensory neurons were depicted in the hypostome. No clear justification for classifying these different populations of cells as ganglionic or sensory was given in the study.

More recently, a single-cell RNAseq study on different *H. symbiolongicarpus* polyp types identified five connected clusters that were labeled as neurons, but according to their patterns in the cellular atlas and the lineage reconstruction presented in the study, most of these clusters are likely developmental stages of neurons rather than distinct clusters representing fully differentiated neuron types (Salamanca-Díaz et al. 2025). *RFamide* is shown as the main maker gene of all neurons in the study, and spatial expression patterns were not generated for any additional markers of these neuron clusters, so further study is needed to verify what these clusters represent. It is likely that there are some neurons or neuron subtypes that have not yet been visualized with modern techniques. To date, no pan-neural marker for *Hydractinia* neurons has been identified, which would highlight the entire nervous system and reveal any unappreciated diversity within this broad cell type. In a recent study of the *Hydra* nerve net (Keramidioti et al. 2024), the authors utilize a newly developed pan-neural antibody (PNab). Future work focused on testing this antibody in *Hydractinia* would generate a more complete view of the ectodermal nerve net and potentially help inform how new neurons are added during growth and regeneration.

**Fig. 3:**
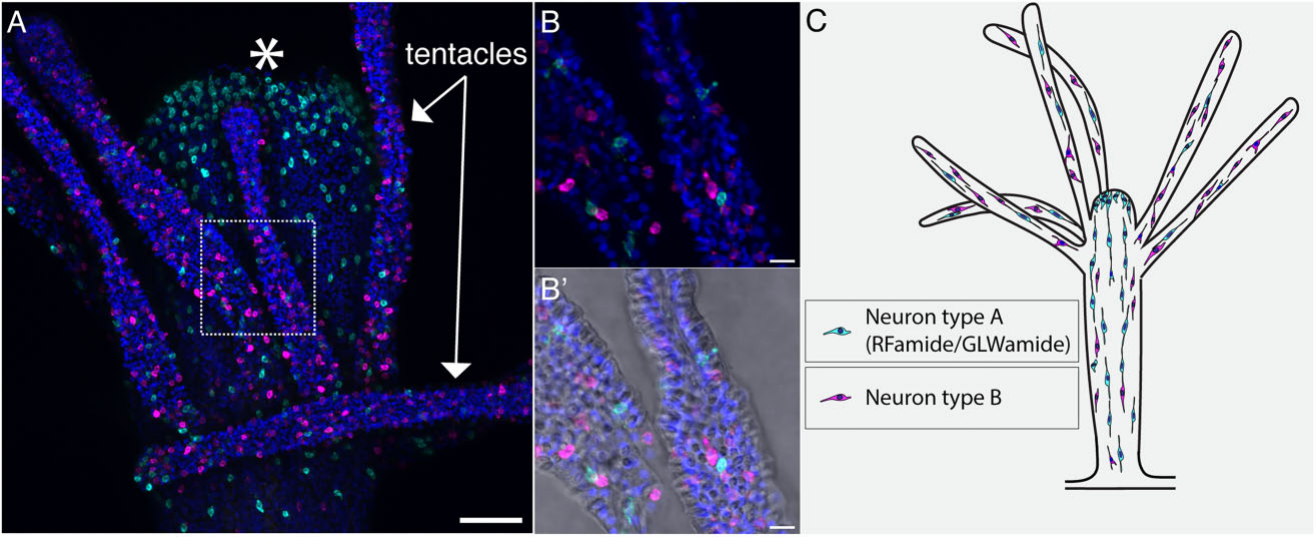
Neurons in the *Hydractinia* feeding polyp. (A) Double HCR-FISH of two mature neuron markers shows spatial location in the oral half of a feeding polyp. (B) Higher magnification image of two tentacles showing non-overlapping expression in two neural subtypes. (B’) Zoom-in showing the two neuron markers together with DIC to highlight their association with nearby cnidocytes. *RFamide^+^* cells (Neuron type A) are shown in cyan, HyS0049.55^+^ cells (Neuron type B) are shown in magenta, and nuclei stained with Hoechst are shown in dark blue. Scale bars are 50 µm in A and 10 µm in B and B’. Asterisk in A marks the mouth. (C) Schematic showing basic distribution of the two neuron types in a *Hydractinia* feeding polyp.

Using our single cell atlas, we were able to identify two separate clusters of potential neurons (Song et al. 2025). Our single-cell transcriptomic data provides some insights into the two neuron clusters. *Elav* (HyS0085.53), which is known to be a pan-neural marker in many animals (Sprecher 2022), was expressed in both clusters. We found that *RFamide*- and *GLWamide*-expressing cells were localized to only one of these two clusters. We selected *RFamide* (HyS0013.338) as a marker gene for the first neuron cluster, and HyS0049.55, a putative neuropeptide, as a marker gene for the second cluster and performed double HCR-FISH on *H. symbiolongicarpus* adult feeding polyps. Both genes, however, were not expressed in all cells in their respective clusters on the single-cell atlas. In feeding polyps, we saw a non-overlapping pattern of cells expressing these markers (Fig. 3A and B’). Consistent with previous studies (Schmich et al. 1998; Chrysostomou et al. 2022), we found many *RFamide*^+^ cells concentrated in the hypostome and fewer in the tentacles. In contrast, HyS0049.55 was primarily expressed in the tentacles, absent from the hypostome, and present but rare in the body column of feeding polyps (Fig. 3A and B’). We are currently calling these two types: Neuron type A (marked by *RFamide* HyS0013.338 and *GLWamide* HyS0009.155) and Neuron type B (marked by HyS0049.55). A schematic showing the overall pattern of these two neuron types in *Hydractinia* feeding polyps is shown in Fig. 3C. Since the Neuron type B cluster has not been previously described, we performed gene ontology (GO) term enrichment analysis on the cluster genes and found enriched terms that could support functions related to neuronal communication including ionotropic glutamate receptor signaling (GO:0035235), ligand-gated ion channel signaling (GO:1990806), and glutamate receptor signaling pathway (GO:0007215). A fibroblast growth factor receptor (FGFR; HyS0036.6) was found to be expressed almost exclusively in the Neuron type B cluster. Further study is required to determine if this gene is related to FGFRs known to be associated with the nervous system in mammals and serving a similar function (Reuss and von Bohlen und Halbach 2003).

In the *Hydra vulgaris* single cell atlas, the distinct neural clusters identified relate both to their oral-aboral position in the animal and whether they are either sensory or ganglionic neurons (Siebert et al. 2019). Therefore, we could hypothesize that our two clusters similarly represent distinct sensory and ganglionic neuron types. However, based on our double HCR-FISH results, we were unable to distinguish whether Neuron type A and B cells correspond to “ganglionic” or “sensory” types or whether there is a mixture of ganglionic and sensory cells in each type. Previous studies in *Hydractinia* and *Hydra* have indicated a mixture of *RFamide*^+^ sensory and ganglionic cells in the hypostome (Klimovich and Bosch 2018; Chrysostomou et al. 2022). Thus, it seems likely that at least our type A cells may represent a mixture of both ganglionic and sensory cells. Performing subclustering analysis of the neural clusters might further split our non-overlapping neuron types into discrete sensory and ganglionic subtypes—for example, the neurons that are primarily located in the tentacles (Neuron type B) may be further split into one or more sensory and/or ganglionic clusters. This would be reminiscent of what has been observed in *Hydra*, where there are four separate clusters of ectodermal neurons located in the tentacles; one cluster consists of sensory neurons, while the other three consist of ganglionic neurons (Siebert et al. 2019). Furthermore, in a recent publication investigating neural activity in *Hydra* using calcium imaging of transgenic animals, Dupre and Yuste identified three neural circuits (CB, RP1, RP2) involving distinct populations of neurons, where firing of a particular circuit elicited a unique behavior (Dupre and Yuste 2017). Linking data from the single-cell atlas of *Hydra* to this work on neural circuits showed that the different neural subclusters in the single-cell atlas are not solely formed by neurons from a single neural circuit. Rather, neurons from multiple different clusters contribute to form each functional circuit (Siebert et al. 2019).

We have observed a wide array of different behaviors in *Hydractinia* feeding polyps, such as longitudinal contractions that lengthen the polyp body; polyp “bending,” where the head and tentacles bend downwards to one side; radial contractions, where the polyps squeeze inwards; and hypostome eversions and feeding, where the tentacles first capture prey and then the tentacles bend to bring the prey to the mouth. Colonies of *Hydractinia* have also been observed to display coordinated responses across the colony when responding to external stimuli (Josephson 1961). The distinct neural circuits that act within the ectodermal nerve net of *Hydractinia* to coordinate any of these complex behaviors are not known, nor is it known how the clusters we have uncovered in our single-cell RNA-seq analysis relate to their behavior. One previous study mentioned that the application of GLWamides in *H. echinata* leads to the contraction of the hypostome and tentacles or even the whole polyp (Schmich et al. 1998), and another study of *Hydra* and the anthozoan *Anthopleura fuscoviridis* has shown that exogenous GLWamides can influence contraction status (Takahashi et al. 1997). Additional study of both the detailed cellular structures of the cells in these clusters and functional studies determining what roles they play will be necessary to make further conclusions.

Battery cell structures have been defined as a functional complex between an ectodermal epithelial cell, 10–20 cnidocytes, and a sensory nerve cell in the tentacles of *Hydra* that are under neural control of the animal (Hobmayer et al. 1990). From our HCR-FISH experiments, we did not find clear evidence of battery cell structures in the tentacles of *Hydractinia* (Fig. 3). We based this on the distribution of our type A and B neurons together with cnidocytes, where there was no clear pattern of cells marked by our type A or B neuron marker and cnidocytes (seen in DIC) in tentacles (Fig. 3B’). We did observe instances of a single type A and a single type B neuron in close proximity with each other (Fig. 3B and B’), similar to a description found in Stokes 1974 that “sensory cells and ganglion cells are often closely apposed in the gastrozooid, especially in the tentacles.” Further studies of the association of cells in *Hydractinia* tentacles, such as those described in *Hydra*’s battery complexes (Hobmayer et al. 1990; Holstein 2012) may be warranted to verify the lack of battery cell complexes or similar structures.

## Summary

*Hydractinia* has been the subject of a long history of research, stretching from the 1800s to the present day. It is attractive as a model organism for many reasons—its population of adult pluripotent stem cells (i-cells), and its tractability in the laboratory, allowing for the study of various aspects of its biology, including the processes underlying allorecognition, coloniality, regeneration, cell-type differentiation, and development—all in a single organism. We have reviewed previous studies on sensory cells in *Hydractinia* and other cnidarians and show that single-cell transcriptomic data are providing an updated and more detailed view of all its cell types, including sensory cell types such as cnidocytes and sensory neurons. Studies of new cell-type markers are providing a unique perspective on these cells based on spatial gene expression profiles, allowing for new insights into their organization within the colony, and revealing potential functions that will need to be tested. Along with current investigations of many aspects of its unique biology, *Hydractinia* is poised to become a model for cnidarian sensory biology research. We are now able to more fully examine and characterize the cnidocytes and neurons of *Hydractinia* by exploring cell-type expression profiles, targeting cell-type markers experimentally, and using these data to inform the evolution of sensory genes in this taxon.

## Materials and methods

A more detailed version of the materials and methods shown here can be found in Song et al. (2025).

### High DAPI staining of nematocytes

Young colonies of *Hydractinia symbiolongicarpus* were relaxed in 4% MgCl_2_: H_2_O before being fixed in 4% PFA: PBS in the presence of 10 mM EDTA for 1–2 h at room temperature. Samples were then washed in 1x PBS + 0.1% Tween-20 (PTw) before being incubated in 143 μM DAPI (Sigma, D9542) for 90 min at room temperature. Samples were washed extensively in PTw, mounted in 70–80% glycerol: PBS and imaged using a Zeiss LSM 710 confocal microscope. Z‐stack projections were generated using Fiji (Schindelin 2012).

### Double HCR fluorescent *in situ* hybridization (dHCR-FISH)

DNA probe sets for genes of interest were designed using the Ӧzpolat Lab probe generator (Keuhn 2022), and probes (DNA oPools™ Oligos) were ordered from Integrated DNA Technologies (IDT). All buffers and hairpin amplifiers were ordered from Molecular Instruments, Inc. (Choi 2018). Following relaxation in 4% MgCl_2_: H_2_O and fixation in 4% paraformaldehyde (PFA) in PTw for 1–2 h, adult feeding polyps were dehydrated into 100% methanol and stored at −20°C for at least 2 h. Following rehydration, samples were washed several times in PTw, incubated in 50% PTw:50% probe hybridization buffer for 15 min at room temperature, and subsequently incubated in 100% probe hybridization buffer for 1 h at 37°C. Gene-specific probe sets were added to a final concentration of 20 nM, and were hybridized for 18–20 h at 37°C. After hybridization, samples were washed in prewarmed wash buffer 4 × 15 min at 37°C, followed by 3 × 5-min washes with 5x SSCT (5x SSC, 0.1% Tween-20) at room temperature. Samples were then incubated in amplification buffer for 30 min at room temperature. Hairpins were prepared by adding 6 pmols of each (h1 and h2) into separate 0.5 mL tubes and heated to 95°C for 90 s before being allowed to cool to room temperature for 30 min. Finally, hairpin pairs were combined, and amplification buffer added to create a “hairpin solution” with a final volume of 100 μL. Samples were incubated overnight in hairpin solution at room temperature before being washed in 5x SSCT for 2 × 5 mins, 2 × 30 mins, and 1 × 5 mins. Samples were counterstained in Hoechst 33342 (ThermoFisher H1399), mounted in 70–80% ultrapure glycerol, and imaged with a Zeiss LSM 710 confocal microscope (Zeiss, Gottingen, Germany). Z‐stack projections were generated using Fiji (Schindelin 2012).

### Creation of a revised single-cell atlas of *Hydractinia* adult feeding polyps

*Hydractinia symbiolongicarpus* feeding polyps were dissected from their colony and washed in calcium- and magnesium-free seawater (CMFSW: 450 mM NaCl, 9 mM KCl, 30 mM Na_2_SO_4_, 2.5 mM NaHCO_3_, 10 mM Tris-HCl, 2.5 mM EGTA, 25 mM HEPES), before being incubated in 1% pronase (Santa Cruz Biotechnology, cat # sc-264144) in CMFSW for 1.5 h on a rocker at room temperature. Once the tissue was fully dissociated, the cell suspension was filtered using a 70 μM Flowmi tip filter (Bel-Art, cat # H13680-0070) and centrifuged at 300 g for 5 min at 4°C. The cell pellet was resuspended in CMFSW and centrifuged again before the cell pellet was resuspended in 200 µL CMFSW. Cells concentration was determined using a hemocytometer. Cells were fixed in either 100% methanol or ACME solution (13:3:2:2 ratio of DNase/RNase-free distilled water, methanol, glacial acetic acid, and glycerol) (Garcia-Castro 2021). Fixed cells were moved to −20°C for storage before library preparation and sequencing. Fixed single cell samples were diluted to 1000 cells/μL and shipped on dry ice to the National Institute of Health Intramural Sequencing Center (Bethesda, MD), where cells were thawed, spun, resuspended, and loaded onto the 10X Genomics platform for encapsulation, with the capture target of 6000–9000 cells per sample. Sequencing libraries were prepared according to the standard 10X Genomics V3 chemistry protocol. cDNA libraries were pooled and sequenced as 150 bp paired-end reads and single indexed on an Illumina NovaSeq6000 with 63 million projected clusters per sample. Raw sequencing data were processed with the CellRanger v7 pipeline (10X Genomics), using default parameters. Preliminary QC was conducted in CellRanger, and samples of low quality were discarded. In addition to the methanol-fixed and ACME-fixed samples, we also included a previously published live single-cell dataset of *Hydractinia symbiolongicarpus* in our analyses (Schnitzler 2024). All count matrices were individually processed and cleaned using Seurat version 4.1.3 (Stuart 2019; Hao 2021) in R. After the initial filtering, the dataset was run through a standard Seurat analysis pipeline using the following parameters: data were normalized, and variable features were selected by running “*SCTransform*,” *vst.flavor = “v2”* (Choudhary 2022). The top 50 principal components were calculated with the *RunPCA* function. Clustering was performed by running the *FindNeighbors* function with dims = 1:15 and then running *FindClusters* with resolution = 0.5. Nonlinear dimensionality reduction was performed to represent the data in a 2D space using uniform manifold approximation and projection (UMAP) (McInnes 2018). Canonical correlation analysis was chosen to integrate the different datasets (Butler 2018). We selected 3000 genes by running *SelectIntegrationFeatures* and integrated datasets by running *IntegrateData*, normalization.method = “SCT.” The integrated dataset was then processed using the standard Seurat pipeline above, with 50 principal components, dims = 1:20 in clustering, and resolution = 0.3 in UMAP. Differential expression (DE) analyses were identified with the *FindAllMarkers* function, with min.pct = 0.3, logfc.threshold = 1. Clusters were annotated based on the DE gene list and known cell type markers.

### GO term enrichment analysis

The R package topGO v.2.54.0 (Alexa and Rahnenfuhrer 2024) was used to perform and visualize Gene ontology (GO) enrichment analysis, and the corresponding GO term accessions retrieved from a customized text file. Enrichment tests were performed using the arguments algorithm= “classic,” statistic=“fisher.”

## Data Availability

Gene specific information for all *Hydractinia* gene IDs discussed in the manuscript can be found at the *Hydractinia* Genome Project Portal https://research.nhgri.nih.gov/hydractinia/.

## Notes

From the symposium “Cnidarian sensory systems as comparative models for the evolution of complexity” presented at the annual meeting of the Society for Integrative and Comparative Biology, January 3-7th, 2025.
